# Assessment of Generative Adversarial Networks Model for Synthetic Optical Coherence Tomography Images of Retinal Disorders

**DOI:** 10.1167/tvst.9.2.29

**Published:** 2020-05-27

**Authors:** Ce Zheng, Xiaolin Xie, Kang Zhou, Bang Chen, Jili Chen, Haiyun Ye, Wen Li, Tong Qiao, Shenghua Gao, Jianlong Yang, Jiang Liu

**Affiliations:** 1 Department of Ophthalmology, Shanghai Children's Hospital, Shanghai Jiao Tong University, Shanghai, China; 2 Cixi Institute of Biomedical Engineering, Ningbo Institute of Materials Technology and Engineering, Chinese Academy of Sciences, Ningbo, Zhejiang, China; 3 School of Information Science and Technology, ShanghaiTech University, Shanghai, China; 4 Joint Shantou International Eye Center of Shantou University and the Chinese University of Hong Kong, Shantou University Medical College, Shantou, Guangdong, China; 5 Department of Ophthalmology, Shibei Hospital, Shanghai, China; 6 Department of Computer Science and Engineering, Southern University of Science and Technology, Shenzhen, Guangdong, China

**Keywords:** optical coherence tomography, retinal disorders, deep learning, generative adversarial networks

## Abstract

**Purpose:**

To assess whether a generative adversarial network (GAN) could synthesize realistic optical coherence tomography (OCT) images that satisfactorily serve as the educational images for retinal specialists, and the training datasets for the classification of various retinal disorders using deep learning (DL).

**Methods:**

The GANs architecture was adopted to synthesize high-resolution OCT images trained on a publicly available OCT dataset, including urgent referrals (37,206 OCT images from eyes with choroidal neovascularization, and 11,349 OCT images from eyes with diabetic macular edema) and nonurgent referrals (8617 OCT images from eyes with drusen, and 51,140 OCT images from normal eyes). Four hundred real and synthetic OCT images were evaluated by two retinal specialists (with over 10 years of clinical retinal experience) to assess image quality. We further trained two DL models on either real or synthetic datasets and compared the performance of urgent versus nonurgent referrals diagnosis tested on a local (1000 images from the public dataset) and clinical validation dataset (278 images from Shanghai Shibei Hospital).

**Results:**

The image quality of real versus synthetic OCT images was similar as assessed by two retinal specialists. The accuracy of discrimination of real versus synthetic OCT images was 59.50% for retinal specialist 1 and 53.67% for retinal specialist 2. For the local dataset, the DL model trained on real (DL_Model_R) and synthetic OCT images (DL_Model_S) had an area under the curve (AUC) of 0.99, and 0.98, respectively. For the clinical dataset, the AUC was 0.94 for DL_Model_R and 0.90 for DL_Model_S.

**Conclusions:**

The GAN synthetic OCT images can be used by clinicians for educational purposes and for developing DL algorithms.

**Translational Relevance:**

The medical image synthesis based on GANs is promising in humans and machines to fulfill clinical tasks.

## Introduction

Optical coherence tomography (OCT), which typically uses near-infrared light to capture high-resolution retinal images in vivo,^1^ is now a standard of care for guiding the diagnosis and treatment of some of the leading causes of blindness worldwide, including age-related macular degeneration (AMD) and diabetic macular edema (DME).^2^^,^^3^ Given the increasing prevalence of these diseases, deep learning (DL) algorithms has been proposed as an alternative screening tool to rectify the manpower and expertise shortage.^4^^–^^6^ Kermany et al.^7^ recently demonstrated highly accurate DL algorithms for OCT imaging classification and performance is comparable to that of human experts.

Despite these promising results, DL algorithms require large, diverse, and well-balanced image training datasets with labels defining structures.^8^ For example, Kermany et al.^7^ trained a DL algorithm using a training dataset (hereinafter referred to as “Cell testing dataset”) with a total of 108,312 images by sharing data from different centers. Such approach has several limitations. First, when data are to be shared between different centers, regulations and state privacy rules need to be considered. As defined by the US National Institute of Standard and Technology, biometric data, including retina images, are personally identifiable information and could possibly be protected from inappropriate access regardless of the original individual study participant consent or local institutional review board (IRB) permission.^9^ Moreover, larger datasets do not necessarily enhance the performance of a DL algorithm. For example, adding large amounts of unbalanced data, such as images from healthy subjects, will most likely not improve performance.

To address these disadvantages, several authors suggested using generative adversarial networks (GANs) to synthesize new images from a training dataset of real images.^10^ Using the Age-Related Eye Disease Study dataset of 133,821 fundus images, Burlina et al.^11^ generated a similar number of synthetic images to train a DL model. They reported DL models trained with only synthetic images showed performance nearing that resulting from training on real images.^11^ Therefore the aim of this study was to assess whether a GAN neural network could synthesize realistic OCT images that satisfactorily serve as training datasets for DL algorithms and education images for retinal specialists.

## Methods

### Datasets

Approval was obtained from the IRB of Shanghai Children's Hospital (identifier, 2018RY029-E01) and Shanghai Shibei Hospital (SSH) (identifier, YL_201805258-05) to conduct the study in accordance with the tenets of the Declaration of Helsinki. Informed consent was not required because of the anonymized usage of images and the retrospective study design.

In this study, we used OCT images from the Kermany et al.^7^ datasets (Cell testing dataset). The detail of this study and Cell's testing dataset has been described previously.^7^ Briefly, a DL model was trained using a dataset with a total of 108,312 images after a tiered grading system. The whole training dataset included 37,206 OCT images from eyes with choroidal neovascularization (CNV), 11,349 OCT images from eyes with DME, 8617 OCT images from eyes with drusen, and 51,140 OCT images from normal eyes, respectively. The study further categorized images with CNV and DME as “urgent referrals,” and drusen and normal as “nonurgent referrals.” In Kermany et al.,^7^ 1000 images (250 from each category) were used as a local validation set. To test the generalization of synthetic OCT images and DL models, we collected a second clinical testing set from the Department of Ophthalmology at SSH from July 2018 to February 2019 (hereinafter referred to as “SSH testing dataset”). Three senior independent retinal specialists (CZ, CJL, and TQ, each with over 20 years of clinical retinal experience) were invited to grade the images. Five sets with 200 images (50 from each category) taken from the Cell testing dataset were used in training the graders. The results of graders were compared with ground true label, and graders passed the training when they achieved an unweighted k value of 0.75 or more in any test set. After searching local electronic medical record databases, a total of 278 OCT images were downloaded using a standard image format based on the manufacturer's instructions (Heidelberg Spectralis; Heidelberg Engineering, Germany). For each patient, only one image most representative of the disease was chosen. As mentioned in the Kermany et al.^7^ study, we chose foveal slice if possible. The clinical dataset comprised 130 OCT images with urgent referrals (CNV and DME), and 148 OCT images with nonurgent referrals (drusen and normal), respectively.

### Development and Evaluation of GAN Synthetic OCT Images

We adopted progressively grown GANs (PGGANs) to synthesis high-resolution OCT images.^12^ GANs consist of a discriminator network (D) and a generative network (G), which is trained by an adversarial learning strategy. With the adversarial learning between the G and D, the G is promoted to generate artificial images bearing greater similarity to real images. PGGANs is an extension to the GAN training process, and achieves high-resolution synthetization (in this study, 256 × 256 pixels) by alternating training and adding new networks G and D. To generate OCT images with correct anatomic structures, a sketch guidance modules G that contains the edge and detailed information was added to growing networks G.^13^ With each additional layer, the resolution is increased (e.g., from 4 × 4 to 8 × 8) allowing the generation of higher-resolution images. All of the GAN development and experiments were conducted using PyTorch (version 1.0, Facebook, USA). The GANs were trained on an Ubuntu 16.04 operation system (Canonical Ltd., UK) with Intel Core i7-2700K 4.6-GHz CPU (Intel, USA), 128-GB RAM, and NVIDIA GTX 1080Ti 12 GB GPU (NVIDIA, USA). In this study, GANs took approximately 40 hours to generate 100,456 synthetic OCT images, including 48,751 images as urgent referrals and 51,705 images as nonurgent referrals, respectively.

To assess whether synthetic OCT images appear realistic to human experts and can be used for clinical evaluation, 400 OCT images (equal numbers of real and synthetic images) were evaluated by two human experts to assess image quality and real versus synthetic. Human experts included two retinal specialists who had more than 10 (retinal specialist 1; HYY) and 20 (retinal specialist 2; WL) years of clinical experience, respectively. First, they were asked to determine whether the image quality was sufficient for clinical grading. The OCT image quality is considered to be good if complete structure of the retina can be observed and is useful for diagnosis. The poor quality of an OCT image is defined as total or partial loss of retinal signal (signal-shield), or the retinal structure cannot be fully displayed (off-center).^14^ Once completing image quality evaluation, the graders were informed that the image set was composed of a mixture of real and synthetic images and were asked to determine whether the image was real or synthetic. Finally, the graders were asked to grade each image as urgent referrals or nonurgent referrals in the two testing datasets (Cell and SSH testing datasets).

A possible concern was how the GAN generated images that are different from training images, or even just copied and pasted original training images. To evaluate performance of GANs at image generation, Fréchet Inception Distance (FID) was used to capture the similarity of generated images to real images, as the FID is a method to compare these statistics.^15^ We calculated FID among four different datasets, including the SSH testing dataset, 200 GAN-generated images, 200 real images (directly copied from the Cell testing dataset), and the Cell testing dataset. A lower FID indicated better similar images.

### Evaluation of GAN Synthetic OCT Images Used for DL Classification

To assess whether synthetic OCT images can be used as a training dataset for DL model, we evaluated diagnostic performance of urgent versus nonurgent referable classification by comparing two DL models trained on real (DL_Model_R) and synthetic (DL_Model_S) OCT images, respectively. Transfer learning with fine-tune technique was adopted to build the DL models by using a modified Inception V3^16^ architecture with weights pretrained on ImageNet.^16^^,^^17^ After removing the final classification layer from the network, we further retained DL models with real and synthetic OCT images independently. DL models were implemented in Tensorflow framework (Google, version 1.10.0) with Keras API (version 2.2.4). All images were resized to 299 × 299 pixels as required by Keras' API. Data augmentation were performed to increase the amount and type of variation within the training dataset, including horizontal flipping, rotation of 10°, and sharpening and adjustments to saturation, brightness, contrast, and color balance. Training was then performed by a stochastic gradient descent in a minibatch size of 32 images with an Adam optimizer learning rate of 0.001. Training was run for 200 epochs, as the absence of further improvement in both accuracy and cross-entropy loss^1^,^2^,^3^,^4^.

**Figure 1.**
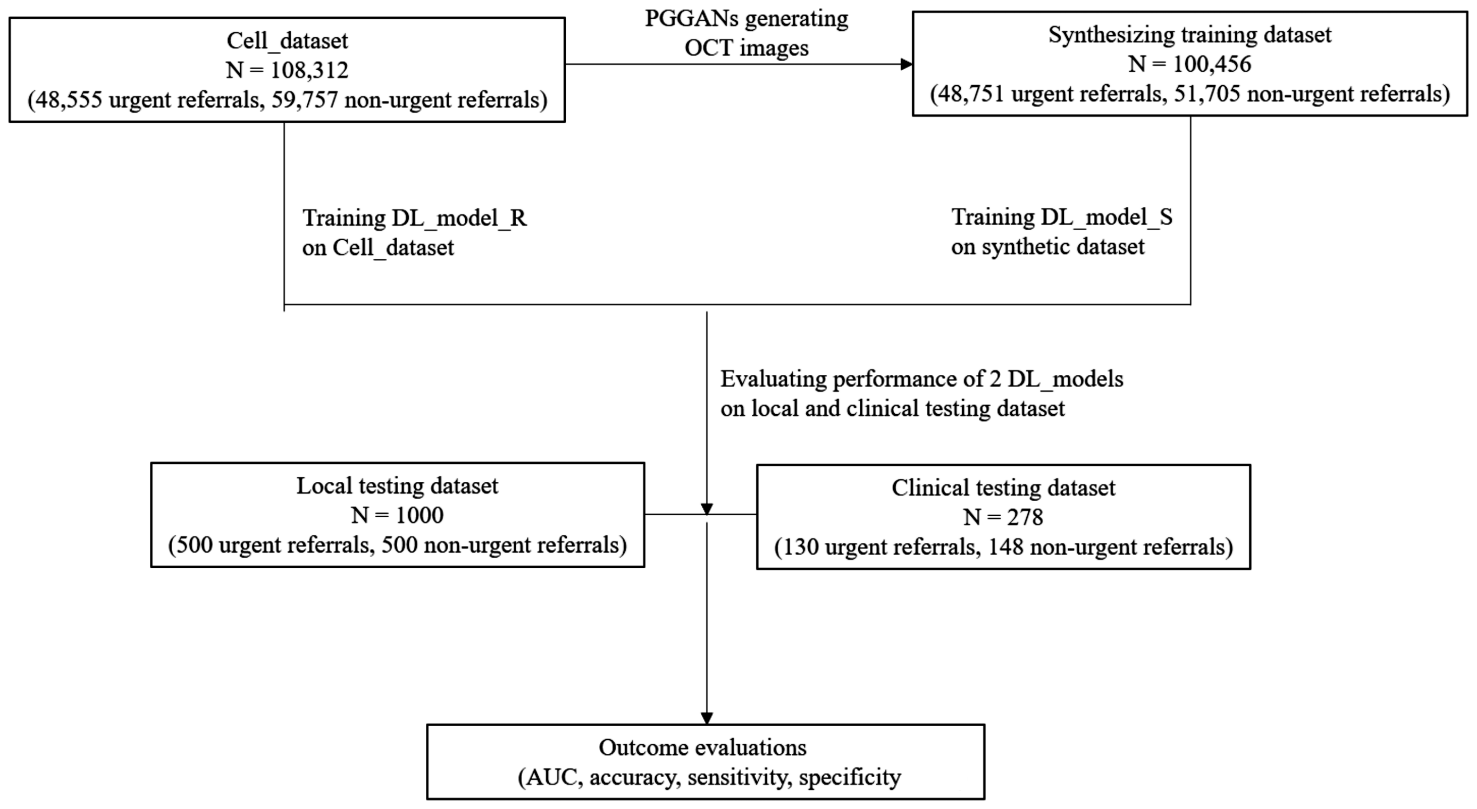
Assessment workflow of using the synthetic OCT images in the classification of various retinal disorders.

**Figure 2.**
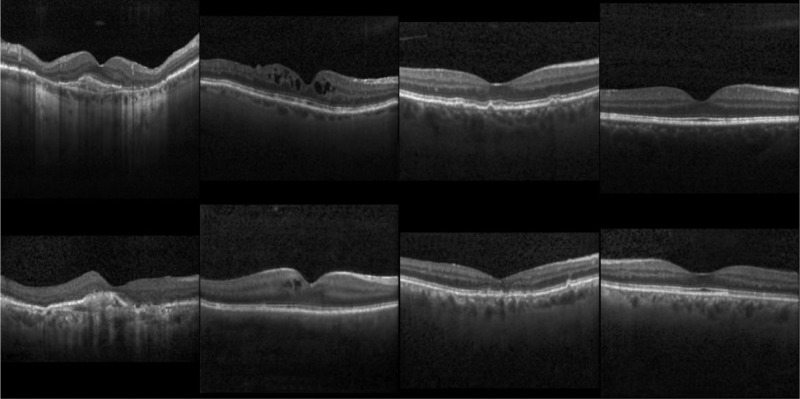
Examples of the synthetic OCT images (the real OCT images are above, and the synthetic OCT images are below).

**Figure 3.**
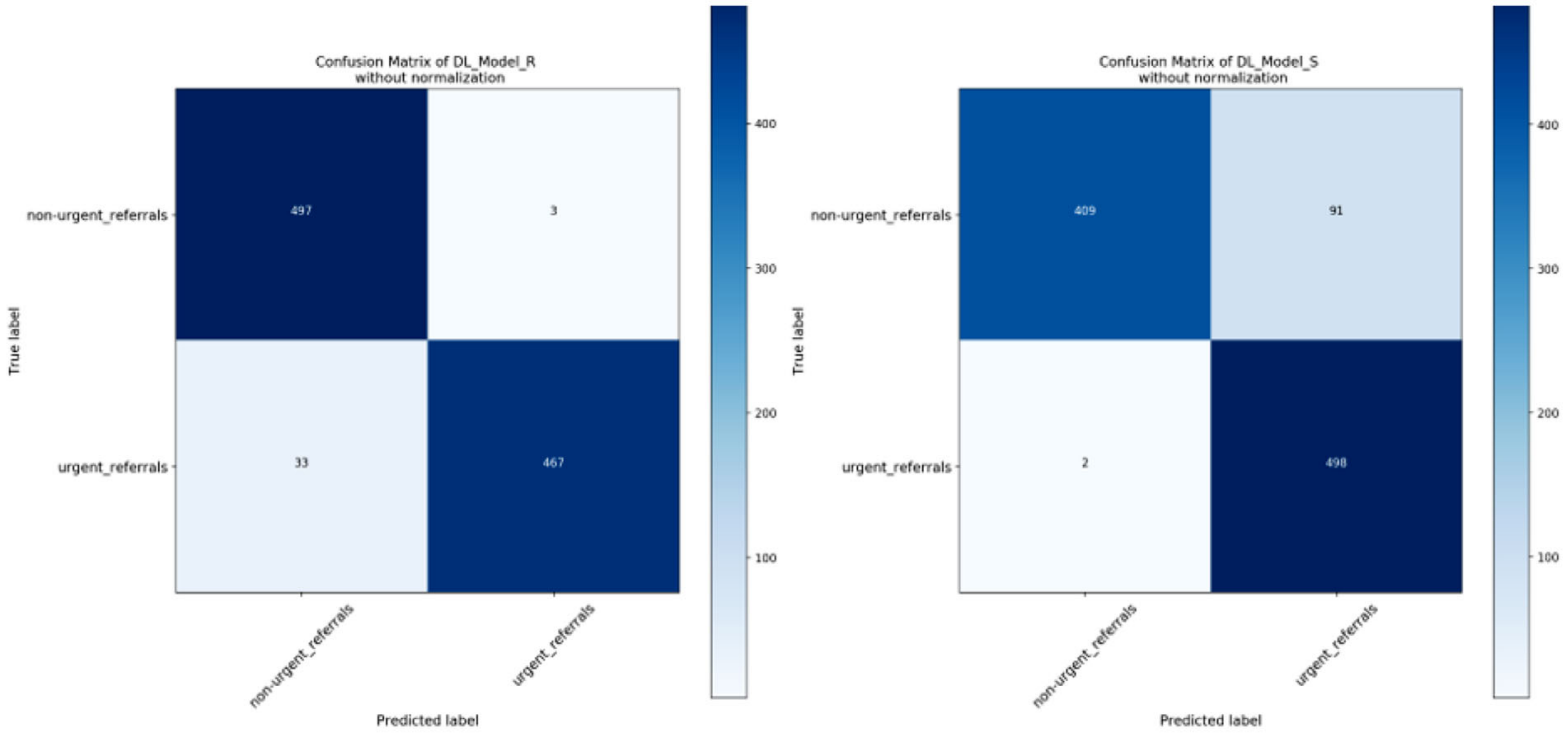
Confusion matrix of two DL models testing in local Cell validation dataset.

**Figure 4.**
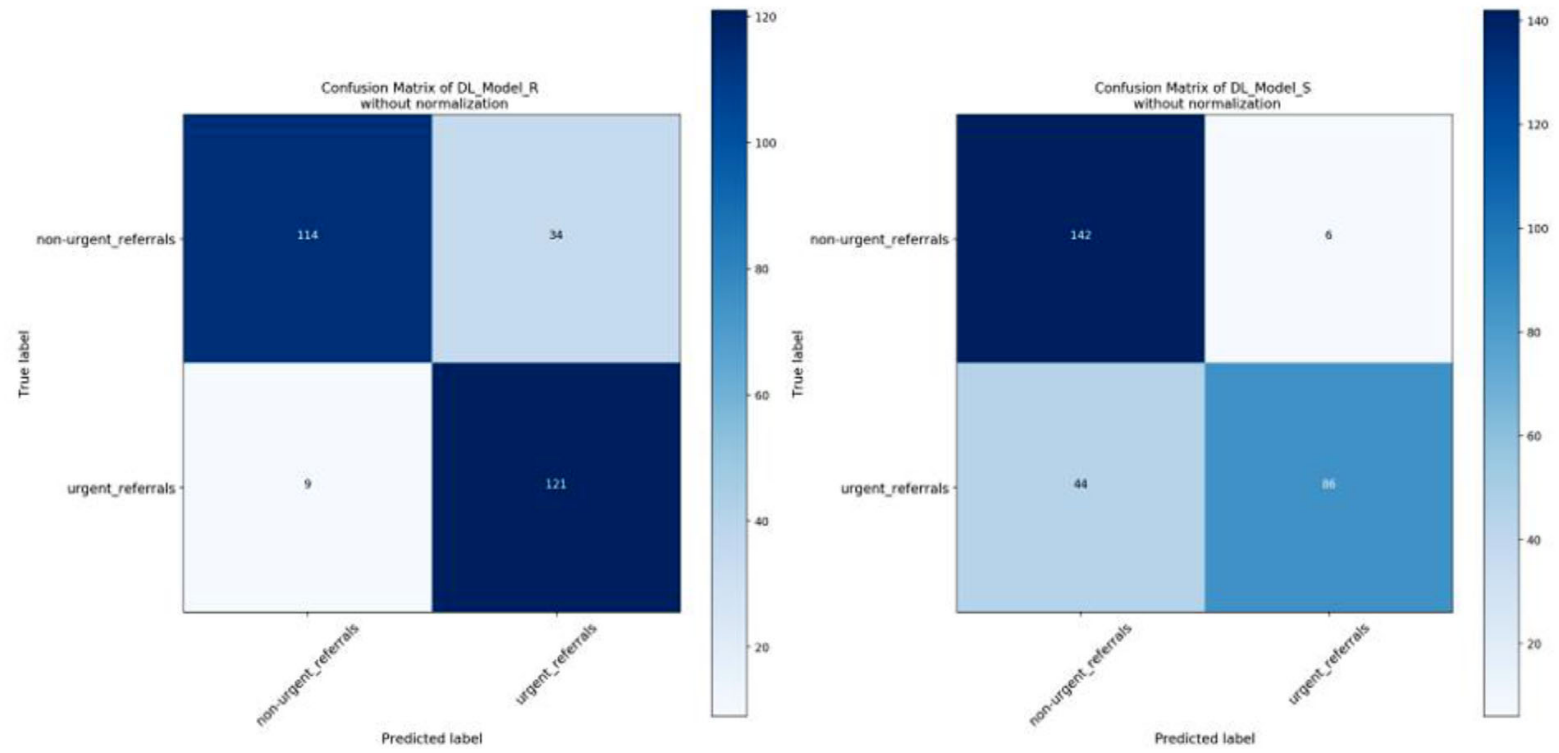
Confusion matrix of two DL models testing in clinical validation dataset.

To test the generalization of DL models, we used two different test datasets. The first local validation dataset was composed of the same testing dataset from the Cell testing dataset (with 1000 images in total), and the second clinical validation dataset was collected from the SSH testing dataset. The DL models’ classification performance in both local and clinical validation datasets were then compared for DL_Model_R and DL_Model_S. To test whether DL model performance could be improved by including GAN generated images, we also trained another two DL models classifying between the SSH testing dataset, but only using the Cell testing dataset during training to compare to results using a dataset combining the Cell testing dataset and a small GAN generated dataset (with 1000 images total). Figure 1 is the assessment workflow of using the synthetic OCT images in the classification of various retinal disorders.

### Statistics

The performance of our algorithm was evaluated in terms of area under the receiver operating characteristic curve (ROC; area under the curve [AUC ]), accuracy, sensitivity, and specificity with two-sided 95% confidence intervals (CIs). The formulas for calculating the accuracy, sensitivity, and specificity were defined as:

(1) $$\mathrm{Accuracy}\;=\;\frac{TP+TN}{TP+TN+FN+FP,}$$

(2) $$\mathrm{Sensitivity}\;=\frac{TP}{TP+FN}\;,$$

(3) $$\text{Specificity}\;=\frac{TN}{TN+FP}\;,$$

where TP, TN, FP, and FN are the true positives, true negatives, false positives, and false negatives, respectively. Intraclass agreement of real and generated image quality for the two retinal specialists was evaluated with kappa statistics (k).

All statistical analyses were performed using the Python and Scikit_learn modules (Anaconda Python, Continuum Analytics, USA).

## Results

### Image Quality and Discrimination Between Real and Synthesizing OCT Images by Human Experts

Calculations based on the Cell testing dataset, FID was 5.49, 10.70, and 60.38 for 200 real images (directly copied from Cell testing dataset), 200 GAN-generated images, and the SSH testing dataset, respectively. Figure 2 demonstrates some examples of the synthetic OCT images. Overall, our approach is capable of generating OCT images that are realistic. The results of image quality graded by two retinal specialists are shown in Table 1^2^. Retinal specialist 1 rated 1.5% of real images to be poor quality versus 2% of synthetic images (k = 0.976). Retinal specialist 2 rated 2% of real images to be poor quality versus 2.5% of synthetic images (k = 0.921). Our results revealed that both real and synthetic OCT images have approximately the same quality for human experts.

**Table 1.** Proportion of Poor Quality for Real and Synthetic OCT Images

	Poor Image Quality (%)
Retina Specialist 1	
All	7 (1.75%)
Real	3 (1.5%)
Synthetic	4 (2%)
Retina Specialist 2	
All	9 (2.25%)
Real	4 (2%)
Synthetic	5 (2.5%)

**Table 2.** Diagnostic Performance of two DL_Models and Retinal Specialists Testing in Cell and SSH Testing Datasets

	Accuracy (95% CI)	Specificity (95% CI)	Sensitivity (95% CI)
A: Testing in Local Cell Validation Dataset			
DL Models			
DL_Model_R	0.96 (0.95–0.98)	0.99 (0.98–1.00)	0.93 (0.91–0.95)
DL_Model_S	0.91 (0.90–0.93)	0.82 (0.80–0.84)	0.99 (0.98–1.00)
Human experts			
Retina specialist 1	0.97 (0.96–0.98)	0.97 (0.96–0.98)	0.98 (0.97–0.99)
Retina specialist 2	0.98 (0.97–0.99)	0.98 (0.97–0.99)	0.97 (0.96–0.98)
B: Testing in Clinical Dataset			
DL Models			
DL_Model_R	0.85 (0.81–0.89)	0.77 (0.72–0.82)	0.93 (0.90–0.96)
DL_Model_S	0.82 (0.78–0.87)	0.96 (0.94–0.98)	0.67 (0.62–0.73)
Human experts			
Retina specialist 1	0.95 (0.93–0.98)	0.93 (0.90–0.96)	0.98 (0.97–1.00)
Retina specialist 2	0.90 (0.86–0.94)	0.87 (0.84–0.90)	0.95 (0.92–0.98)

When comparing discrimination between real versus synthetic OCT images, our results showed that two human experts had limited ability to discern real from synthetic images, with an accuracy of 59.50% (95% CI, 53.5%–65.3%) for retinal specialist 1, and 53.67% (95% CI, 47.8%–59.5%) for retinal specialist 2.

### Performance of DL Classification on Real and Synthesizing OCT Images

**Figure 5.**
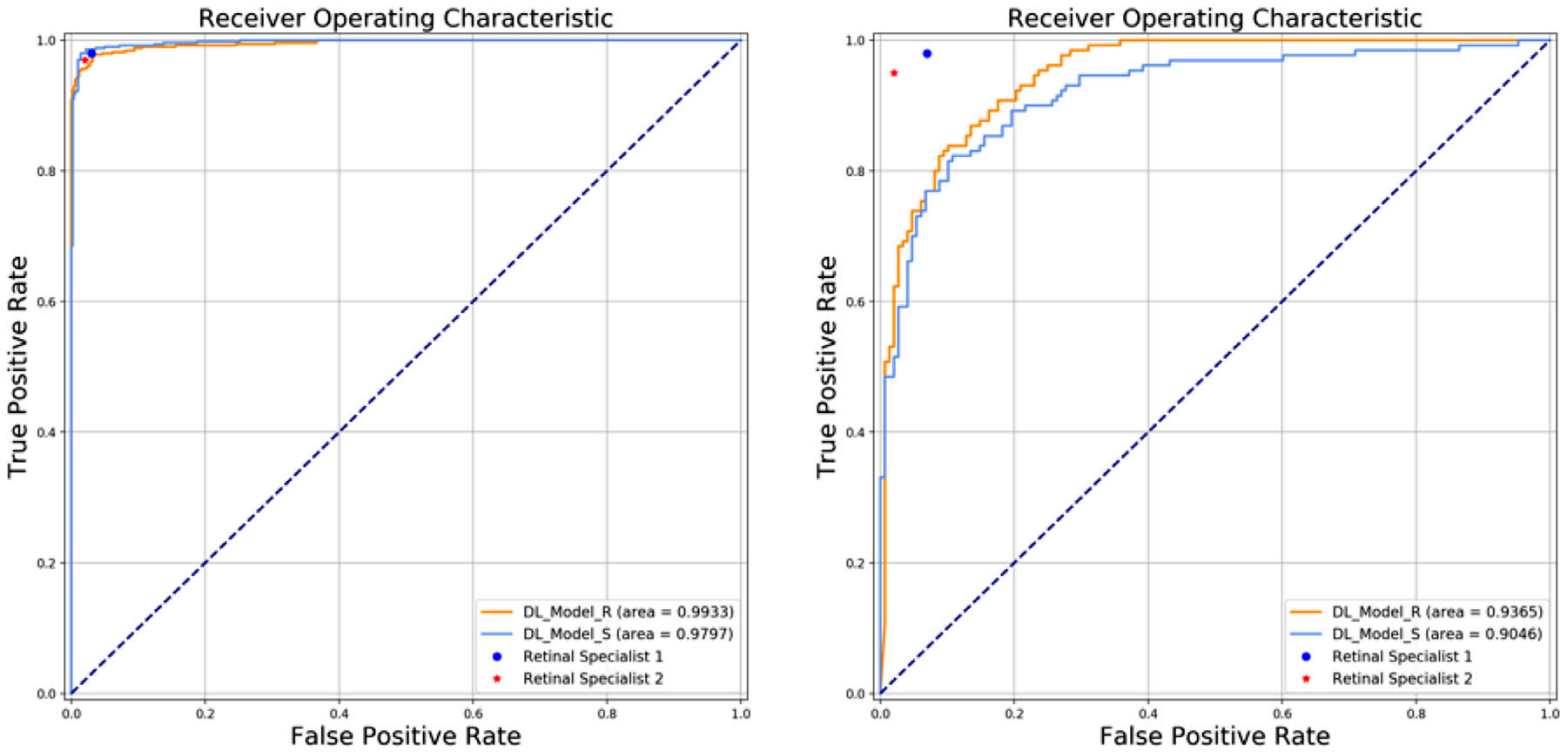
ROC curves of the two DL models tested in the local Cell validation dataset (left) and clinical validation dataset (right).

Figure 3 and Figure 4 are the confusion matrixes of two DL models testing in local Cell validation dataset and clinical validation dataset, respectively. Figure 5 shows the ROC curves. Overall, moderate performance decrease was achieved in both Cell and SSH testing datasets when using the DL model trained only with synthetic OCT images, but the performance was still considered to be good. After adding GAN-generated images into the Cell testing dataset as the DL model training dataset, the AUC was improved from 0.84 (95% CI, 0.80–0.88) to 0.87 (95% CI, 0.82–0.90) as shown in Table 2.

## Discussion

In this study, we developed and evaluated GANs adopted to generate high-resolution OCT images with urgent and nonurgent referrals. The results suggest that synthetic OCT images can be used by clinicians in place of real images for education or clinical training purposes. Moreover, there was moderate performance decreaseusing the synthetic training dataset, suggesting that synthetic OCT images can also serve as augmentation of training datasets for use by DL models.

DL has made dramatic progress for discriminative medical image analyses tasks and achieved performance exceeding traditional machine learning and close to that of human experts.^6^^,^^18^^,^^19^ However, DL approaches require a large number of high-quality data. An obvious approach is data sharing from different centers. This is often impeded by IRB concerns, patients’ consent, or proprietary data restrictions. Moreover, the implementation of data sharing requires hardware and software investments, expertise, and is labor-intensive. Recently, GANs were proposed to generate synthetic images that matched the real images via an adversarial process. GANs have been successfully applied to many medical image syntheses tasks, including retinal fundus, melanoma lesion, computed tomography, and magnetic resonance images synthesizing.^20^^–^^22^ Burlina et al.^19^ suggested that GANs-synthesized fundus images of AMD are realistic and could be used for both education and for machine training. Using a similar GANs model mentioned in a previous study, our results also show the ability of GANs to synthesize realistic OCT images. This result was encouraging in that the GANs can generate synthetic OCT images with high quality assessed by human experts.

The potential application of this technique is promising, as our study also showed that GAN OCT images can also be served as image augmentation for both clinical purpose and DL models training. Previously, most of DL research groups used data sharing from different centers to increase the number of input data for network training. For detecting glaucomatous optic neuropathy, Li et al.^5^ reported a DL model with AUC of 0.996 training on 6 different eye centers with more than 200,000 fundus images. Using datasets from two different countries, Gulshan et al.^4^ reported AUC of 0.974 for detection of diabetic retinopathy. Recently, Kermany et al.^7^ reported a DL model with AUC of 0.999 for screening common treatable blinding retinal diseases, such as CNV or EMD, using OCT images. This study involved more than 100,000 OCT images in four eye centers from two different countries. It is interesting to note that, using the same Cell testing dataset, our model trained on all-synthetic OCT images achieved a similar AUC of 0.98 with that of the DL model trained on all-real OCT images. Our result suggests that this technique could also hold promise for augmenting DL models, whereas preserving anonymity and obviating constraints owing to IRB or other use restrictions.

It is important to ensure the generalizability of a DL model by testing in independent datasets with clinical settings.^21^ As recently work showing highly accurate DL algorithms for various medical image classification tasks in well-labeled dataset, it is interesting to validate the performance of DL models in real-life. Ramachandran et al.^23^ reported a moderate decrease of AUC (0.901 vs. 0.980) when using a clinical validation dataset for screening diabetic retinopathy. In our study, moderate performance decrease was achieved in two testing datasets when using DL model trained only with synthetic OCT images. Although the performance was still considered to be good, it should be noted that DL_Model_S can only achieve sensitivity of 0.67 when tested in the SSH testing dataset. Therefore caution should be exercised when interpreting results of the DL model.

One limitation of this study was that images synthesized were 256 × 256 pixels, which is lower than that of OCT images used in the original Kermany et al.^7^ study. The default input size for the inception model is 299 × 299 pixels. It is possible for GANs to generate higher resolutions (e.g., 1024 × 1024 or above), however, it requires a longer training time (at least 1 month for the hardware used in the current study), which was impractical for this study. Second, although four classes of OCT images were generated by PGGANs separately, we combined them into two groups to train DL models. It would be more clinically useful if DL models could be trained to do multiple classifications in the future. Third, other retinal disorders, such as macular hole, epiretinal membrane, or pigment epithelium detachment, were not included in the current study as they are less common conditions. Future work will involve generating such rare retinal disorders and might help improve diagnostic accuracy of DL models. Finally, as we used same reference standard as in the Kermany et al.^7^ study to determine the results of retinal specialists’ grading, graders’ diagnostic accuracy may be overestimated due to incorporation bias.

## Conclusions

The GAN synthetic OCT images can be used by clinicians in place of real OCT images for developing DL algorithms. Further studies are warranted to investigate where this technology could be best utilized within clinical and research settings.
